# LINC00894 Regulates Cerebral Ischemia/Reperfusion Injury by Stabilizing EIF5 and Facilitating ATF4-Mediated Induction of FGF21 and ACOD1 Expression

**DOI:** 10.1007/s11064-024-04213-w

**Published:** 2024-07-26

**Authors:** Yifei Chen, Hengxiang Cui, Zhuanzhuan Han, Lei Xu, Lin Wang, Yuefei Zhang, Lijun Liu

**Affiliations:** 1 Department of Emergency and Critical Care Medicine, The Second Affiliated Hospital of Soochow University, No.1055, San Xiang Road, Suzhou, Jiangsu 215004 China; 2 Department of Emergency Medicine, The Affiliated Hospital of Yangzhou University, Yangzhou, Jiangsu 225012 China; 3 Shanghai Key Laboratory of Psychotic Disorders, Brain Health Institute, Shanghai Mental Health Center, National Center for Mental Disorders, Shanghai Jiao Tong University School of Medicine, Shanghai, 200030 China; 4 Department of Emergency Medicine, The Affiliated Hospital of Xuzhou Medical University, Xuzhou, Jiangsu 221002 China; 5 Department of Anesthesiology, The Affiliated Hospital of Yangzhou University, Yangzhou, Jiangsu 225012 China

**Keywords:** LINC00894, Ischemia, Oxygen–glucose deprivation, FGF21, ACOD1

## Abstract

The non-coding RNA LINC00894 modulates tumor proliferation and drug resistance. However, its role in brain is still unclear. Using RNA-pull down combined with mass spectrometry and RNA binding protein immunoprecipitation, EIF5 was identified to interact with LINC00894. Furthermore, LINC00894 knockdown decreased EIF5 protein expression, whereas LINC00894 overexpression increased EIF5 protein expression in SH-SY5Y and BE(2)-M17 (M17) neuroblastoma cells. Additionally, LINC00894 affected the ubiquitination modification of EIF5. Adeno-associated virus (AAV) mediated LINC00894 overexpression in the brain inhibited the expression of activated Caspase-3, while increased EIF5 protein level in rats and mice subjected to transient middle cerebral artery occlusion reperfusion (MCAO/R). Meanwhile, LINC00894 knockdown increased the number of apoptotic cells and expression of activated Caspase-3, and its overexpression decreased them in the oxygen–glucose deprivation and reoxygenation (OGD/R) in vitro models. Further, LINC00894 was revealed to regulated ATF4 protein expression in condition of OGD/R and normoxia. LINC00894 knockdown also decreased the expression of glutamate-cysteine ligase catalytic subunit (GCLC) and ATF4, downregulated glutathione (GSH), and the ratio of GSH to oxidized GSH (GSH: GSSG) in vitro. By using RNA-seq combined with qRT-PCR and immunoblot, we identified that fibroblast growth factor 21 (FGF21) and aconitate decarboxylase 1 (ACOD1), as the ATF4 target genes were regulated by LINC00894 in the MCAO/R model. Finally, we revealed that ATF4 transcriptionally regulated FGF21 and ACOD1 expression; ectopic overexpression of FGF21 or ACOD1 in LINC00894 knockdown cells decreased activated Caspase-3 expression in the OGD/R model. Our results demonstrated that LINC00894 regulated cerebral ischemia injury by stabilizing EIF5 and facilitating EIF5-ATF4-dependent induction of FGF21 and ACOD1.

**Supplementary Information:**

The online version contains supplementary material available at 10.1007/s11064-024-04213-w.

## Introduction

Cerebral ischemia–reperfusion (CI/R) injury during a stroke is a complex pathophysiological process, combined with a rapid cascade reaction. This process commonly induces inflammatory cytokine expression and inflammatory cell infiltration, resulting in inflammatory reactions that aggravate cellular damage in the brain [1–3]. The rodent middle cerebral artery occlusion (MCAO) and oxygen–glucose deprivation/reoxygenation (OGD/R) cell models are commonly used to explore neuropathological mechanisms of ischemic stroke [4].

Previous studies have suggested that oxidative stress could be a crucial pathological factor during ischemia/reperfusion injury, and reactive oxygen species (ROS) at high levels may induce neuronal apoptosis [5]. Cerebral ischemia causes a substantial decrease in the level of the endogenous ROS scavenger glutathione (GSH) in tissues. Mammalian GSH is synthesized from glutamate, cysteine, and glycine by γ-glutamyl-cysteine ligase synthetase including γ-GC modifier (GCLM) subunit, γ-GC ligase catalytic (GCLC) subunit, and GSH synthetase (GSS) [6]. GCLC knockout induces glutathione production loss, which may induce oxidative stress; it is strongly associated with the progression of neurodegenerative disorders [7, 8]. The overexpression of GCLC also inhibits cellular apoptosis, leading to the alleviation of inflammation in acute lung injury [9].

Activating transcription factor 4 (ATF4) is a stress-induced transcriptional factor that controls the transcription of genes involved in autophagy, oxidative response, and nutrient sensing [10–12]. Furthermore, ATF4 reportedly regulates GSH biosynthesis and exercise resistance to oxidative stress [13, 14]. Furthermore, activated ATF4 regulates the expression of fibroblast growth factor 21 (FGF21), a metabolic and stress cytokine typically expressed in liver cells under cellular stress and enhanced cellular stress resistance [15–18]. FGF21 can attenuate age-related metabolic and stress disorders. FGF21 serves as a neuroprotectant with cognition-enhancing effects [19]; it can inhibit neuroinflammation following ischemic stroke [20] and maintain blood–brain barrier integrity in an ischemic stroke model [21].

Eukaryotic translation initiation factor 5 (EIF5) interacts with the 40 S initiation complex, facilitating the hydrolysis of bound GTP with simultaneous binding of the 60 S ribosomal subunit to the 40 S initiation complex. The resulting 80 S ribosomal initiation complex participates in peptidyl transfer and chain elongation [22]. EIF5 regulates scanning by the preinitiation complex and translation of GCN4, which is the yeast ATF4 equivalent [23]. Overexpression of EIF5 induces ATF4 expression in yeast and human cells [24]. Under stress, EIF5 could increase ATF4 translation from non-AUG codons [25]. Furthermore, cyst stem cells lacking EIF5 reportedly showed an imbalance in cell proliferation and apoptosis during spermatogenesis [26].

Cis-aconitate decarboxylase (ACOD1) encoded by immunoresponsive gene 1 (*Irg1*) is an enzyme responsible for the decarboxylation of cis-aconitate from the Krebs cycle in macrophages [27]. It was recently reported that in a MCAO model, the endogenous ACOD1 was protective against cerebral ischemia/reperfusion injury [28]. The mechanism by which ACOD1 catalyzes the production of itaconate to regulate inflammation continues to attract the interest of researchers.

Long non-coding RNAs (lncRNAs) participate in various biological processes such as chromatin and genome architecture remodeling, RNA or protein stabilization, transcription regulation, cell self-renewal and differentiation, and DNA damage response [29–31]. Numerous human lncRNAs have been identified to date; however, less than 3% have been experimentally validated for their functions [32]. Therefore, it is essential to further investigate the biological functions of these endogenous transcripts and their role as signal transducers.

LINC00894 (ENST0000044489) is a non-coding RNA derived from the X chromosome; it regulates the expression of transforming growth factor-beta 2 (TGF-β2) and zinc finger E-box-binding homeobox 1 (ZEB1), which may affect tamoxifen resistance in breast cancer [33]. LINC00894 might have a potential role in the nervous system, because an RNA expression correlation analysis of clinical samples suggested that LINC00894 and EIF5 are co-expressed and may together regulate extracellular matrix receptor signal transduction and long-term potentiation (LTP) dysfunction [34]. LINC00894 may also regulate the expression of G protein-regulated inducer of neurite outgrowth 1 (GPRIN1), which regulates axon growth in hippocampal neurons and synapse formation in brain development [35, 36]. Currently, our understanding of the biological functions of LINC00894 is limited. In this study, we aimed to reveal the role and mechanism of action of LINC00894 in protecting the brain from ischemic injury by identifying its interacting proteins.

## Materials and Methods

### Cell Culture and Transfection

Immortal cell lines were acquired from the American Type Cell Culture Collection (ATCC) and cultured at 37 °C in a humidifier containing 5% CO_2_. Both BE(2)-M17 (CRL-2267) being simply referred to as M17 cells and SH-SY5Y (CRL-2266) cell lines were maintained in Opti-MEM (31,985,062; Gibco, Thermo Fisher Scientific, Waltham, MA, USA) containing 5% fetal bovine serum (FBS; Invitrogen, Carlsbad, CA, USA). PC12 cells were maintained in low-glucose Dulbecco’s modified Eagle medium (DMEM) supplemented with 10% heat-inactivated horse serum (Invitrogen) and 5% FBS (Invitrogen); 293T cells were maintained in DMEM (10,569,044; Gibco). Lipofectamine™ 3000 reagent (L3000015; Thermo Fisher Scientific) was used for the transfection of DNA constructs into cells according to the manufacturer’s instructions.

To obtain primary mouse brain fibroblasts (MBFs), the cerebral meninges samples of fetal mice were peeled under a microscope (Leica S8 APO; Wetzlar, Germany). These were then placed in a Petri dish with precooled phosphate-buffered saline (PBS) and digested with 0.25% trypsin at 37 °C for 20 min; the cell suspension was obtained by agitation. The cells were collected via centrifugation at 200–400 × *g*, and the pellet was resuspended in DMEM containing 10% FBS, 100 units/mL penicillin, and 100 µg/mL streptomycin. Thereafter, approximately 8 × 10^4^/mL cells were seeded in a 3.5-cm Petri dish at 37 °C with 5% CO_2_. The medium was replaced every other day.

### DNA Construct

The full-length DNA (3414 bp) coding LINC00894 (ENST00000449111.5) was amplified using PCR with the following primer pairs: LINC00894-F: 5′-GCG**GCTAGC**ACTTGCCACAAGGAGACGCTG-3′ (Nhe1) and LINC00894-R: 5′-GCG**GCGGCCGC**TCCAAATAGGCACTAAATCCA-3′ (Not1); the fragment containing the Nhe1 and Not1 cloning sites was cloned into pcDNA3.1. EIF5 (NM_001969.5), ATF4 (NM_001675.4), FGF21 (NM_019113.4), and ACOD1 (NM_001258406.2) overexpression vectors were constructed by cloning the three open reading frames into pcDNA 3.1 vectors. CRISPR reagents were generated to target LINC00894 (designed as LINC00894 gRNA) using the following oligo pairs: LINC00894-gRNA4-F: 5′-CACCG**AGGCAGGGTGTGCTGGGTCT**-3′ and LINC00894-gRNA4-R: 5′**-**AAAC**AGACCCAGCACACCCTGCCT**C-3′ (**bases in bold typeface are gRNA sequences**) and cloned into a lentiviral vector (lentiCRISPR; Addgene plasmid # 52,961). qRT-PCR was used to confirm that LINC00894 expression was silenced by LINC00894 gRNA. The FGF21 promoter DNA was amplified using PCR with the following primers: 5′-GACAAGGAGCGTGACCATTGAAGC-3′ and 5′-ATGGCTCGGGTCCTCAGGTGATCT-3′; ACOD1 promoter DNA was amplified using PCR with the following primers: 5′-CATAAGATGCCACAATTTGGTG-3′ and 5′-CGTTGTAAAGAAGAGGTTCAG-3′; the two promoter DNA fragments were cloned into the pGL4.0 luciferase report vector (Promega, WI, USA) using Gibson assembly cloning kits (E5510S, NEB, MA, USA).

### Animals and MCAO/R Model

Healthy adult male Sprague–Dawley rats or male C57BL/6 mice aged 8 weeks were purchased from the Comparative Medicine Center of Yangzhou University, China. All experiments were conducted by following the “Guiding opinions on treating experimental animals” issued by the Ministry of Science and Technology and approved by the Animal Ethics Committee of Yangzhou University (Yangzhou, China; Approval No. 202,311,004 and No. 202,304,026).

Control recombinant adeno-associated virus (AAV) and LINC00894 RNA carrying AAV (pAAV-CMV-PGI-19,090,012-tWPA) were obtained from Heyuan Biotechnology Shanghai Company, China (www.oobio.com.cn). The animals were randomly divided into control and treatment groups, which were administered AAV (AAV-con) and AAV carrying LINC00894 (AAV-LINC00894), respectively. The animal was anesthetized by injecting Zoletil^®^50 into the abdominal cavity before being fastened to a brain stereotaxic apparatus (RWD Life Science, Shenzhen, China). Then the virus were intracerebroventricularly (icv.) injected into the left lateral ventricle of the animal (rats X: +3.0 mm, Y: +1.0 mm, Z: −3.0 mm; mice X: +1.0 mm, Y: +1.0 mm, Z: −2.0 mm); a dose of 2 µl of LINC00894 virus or control AAV virus (titer 7.72 × 10^12^) was injected into rats, while 0.8 µl of these virus (titer 7.72 × 10^12^) was administered to mice. The MCAO/R model was established after a month of virus administration, as previously described [37]. The animal was anesthetized by injecting Zoletil^®^50 (50 mg/kg) into the abdominal cavity, and the anterior midline skin was incised. Thereafter, the internal carotid artery (ICA), external carotid artery (ECA), and left common carotid artery (CCA) were separated. The distal end of the ECA was ligated, and the ECA and its branches were coagulated near the ligation point. A suture was inserted from the ECA through the CCA bifurcation into the ICA, with the arterial clamp on the ICA loosened and the suture inserted into the intracranial ICA segment. The blood flow was blocked for 1 h and the suture was retrieved for reperfusion for 24 h. Neurological behavioral tests were performed 24 h post-reperfusion. Longa neurological examination scores were used to assess neurological deficit, which was divided into six grades: 0 points, no neurological deficit; 1 point, failure to fully extend left forelimb, mild focal neurological deficit; 2 points, circling to the left, moderate neurological deficit; 3 points, falling to the left, severe focal deficit; 4 points, no spontaneous walking and depressed level of consciousness; 5 points, death. The average score was used to compare the behavior difference between LINC00894 virus or control AAV virus infected animal in MCAO model. At last, the infarct size was determined by staining with 2,3,5-triphenyltetrazolium chloride (TTC, Amresco LLC., 298-96-4) and was analyzed with Image J software (v1.50i).

### **Oxygen–Glucose Deprivation** (**OGD/R**)

Cells were seeded into a six-well plate at 50% confluence and maintained in culture medium for 24 h before being washed thrice using PBS; they were then maintained in DMEM without glucose under 95% N_2_ and 5% CO_2_ for 4–16 h. Following oxygen deprivation treatment, the medium was replaced with a normal expansion medium, and the cells were maintained for another 1 h before the cells were used for further analysis. The DNA transfected cells were used 24 h after transfection for OGD challenge or other experiment.

### Apoptosis Detection

Cellular apoptosis was evaluated using fluorescence-activated cell sorting with a PE–Annexin V apoptosis detection kit (cat:559,763, BD Biosciences, San Jose, CA, USA) according to the manufacturer’s instructions. Briefly, cell pellets in each treatment were obtained by centrifugation of the samples at 400 *× g* before being resuspended in binding buffer at 25 °C at a density of 1 × 10^6^ cell/mL; the cell suspension was incubated with annexin-V–FITC and 7-AAD at 25 °C in the dark for 15 min. The cells were then detected using a flow cytometer (CytoFLEX, Beckman, San Jose, CA, USA), and the percentages of apoptotic cells in each group were analyzed using FlowJo (vX 10.0.7r2).

### **RNA–Protein Pull-Down and Electrophoretic Mobility Shift Assay** (**EMSA**)

The DNA construct encoding LINC00894 was synthesized and cloned into pcDNA3.1. Sense and antisense LINC00894 were obtained using PCR before being synthesized using the MEGAscript™ T7 Transcription Kit (AM1333; Thermo Fisher Scientific). The total protein extract from SH-SY5Y was incubated with sense and antisense LINC00894 labeled with biotin using the Pierce™ Magnetic RNA-Protein Pull-Down Kit (#20,164; Thermo Fisher Scientific) according to the manufacturer’s instruction. The sense and antisense LINC00894 bound components were analyzed using mass spectrometry, and the unique peptide sequences enriched in the sense LINC00894 group were analyzed using immunoblotting.

To confirm if sense LINC00894 interacted with EIF5, we synthesized LINC00894 using the MEGAscript™ T7 Transcription Kit (AM1333; Thermo Fisher Scientific); approximately 5 pmol/L LINC00894 was labeled using T4 polynucleotide kinase and [γ32-P] ATP. A 30-µL EMSA reaction mixture containing recombinant EIF5, 100 mM KCl, 1 µg poly (dI–dC), 0.033 mm ZnCl_2_, and ∼40 fmol labeled LINC00894 was incubated on ice for 20 min. Protein**–**RNA complexes were resolved using 5% polyacrylamide gel electrophoresis (PAGE) without sodium dodecyl sulfate at ∼130 V for 2 h at 4 °C; the gels were then dried, and the protein**–**RNA complexes were visualized using autoradiography.

### RNA Immunoprecipitation Assay

For RNA immunoprecipitation, 1.5 × 10^7^ SH-SY5Y cells were washed thrice with cold PBS and scraped into 1 mL of PBS. The cells were then centrifuged and lysed using RIPA buffer (Merck Millipore, MA, USA). The protein A/G magnetic beads were pre-bound with 6 µg EIF5 antibodies or IgG in immunoprecipitation buffer (140 mM NaCl, 20 mM Tris-HCl pH 7.5, 0.05% TritonX-100) for 2 h before being incubated with 100 µL of cell lysates overnight at 4 °C with agitation. The magnetic beads were washed, and the bound RNA was eluted with 400 µL of elution buffer for 2 h. The eluted RNA was precipitated with ethanol and dissolved in RNase-free water. Enrichment of certain fragments from the IgG control or EIF5 groups was determined using real-time PCR with the following primer pair: sense: 5′-AGCAGACCATGAGAGGGAGT-3′, antisense: 5′-CCTCTAGTGGGCAACCCTTG-3′. The antibodies used in this experiment were as follows: EIF5 (Thermo Fisher Scientific; A301-771 A) and Anti-IgG (Cell Signaling Technology, MA, USA; #2729).

### RNA Isolation and qRT-PCR

Cellular total RNA was obtained from cells using the TRIzol reagent (Invitrogen). RNA quality was evaluated using electrophoresis and the ratio of OD 260/OD 280. For cDNA synthesis using reverse transcription with the PrimeScript RT Reagent Kits (Takara Bio, Kusatsu, Japan), 1000 ng of RNA was used. cDNA was used as the template and was amplified in triplicate using qRT-PCR with the CFX Connect Real-Time PCR Detection System (Bio-Rad, CA, USA) and SYBR Premix Ex Taq (Takara Bio) according to the manufacturer’s instructions. All primers used are shown in Table 1. The qRT-PCR cycling conditions were as follows: initial denaturation at 95 °C for 45 s, 95 °C for 35 s, and annealing at 60 °C for 35 s for 40 cycles. The 2^−ΔΔCT^ method, with *β-actin* as the internal control, was used to determine the relative expression. Fluorescent signals were measured after each primer-annealing step at 60 °C.

**Table 1:** Primers used in the qRT-PCR

β-Actin-F	GTACGCCAACACAGTGCTG
β-Actin-R	CGTCATACTCCTGCTTGCTG
LINC00894-F	AGCAGACCATGAGAGGGAGT
LINC00894-R	CCTCTAGTGGGCAACCCTTG
kmo-rat-F	CAATGGCATCGTCGGACACT
kmo-rat-R	CATTGGGGTAGGACTCCACG
aox4-rat-F	CTCAACCCCATTTTGGCAGC
aox4-rat-R	GCGTCACTCAGCATTTGGTC
acod1-rat-F	AACGGTGTTGCTATTCACTCC
acod1-rat-R	TTGGCTGCATTGCCGATATG
fgf21-rat-F	CGAGGCATACCCCATCTCTG
fgf21-rat-R	ACTGTTCCGTCCTCCCTGAT
nkx6-3-rat-F	CTACCTTCACAGGCCACCAG
nkx6-3-rat-R	TCTTCTCGTCGTCCGAGTCT
atf4-rat-F	TGTTGGCGGGGGACTTAATG
atf4-rat-R	AAAAGGCATCCTCCTTGCCG
aqp5-rat-F	CACCATGAAAAAGGAGGTGTGC
aqp5-rat-R	TGTGTTGTTGTTCAGCGCAT
tlr5-rat-F	TCCTTCTCTGGCCATAGGCT
tlr5-rat-R	ACAGTTAGGCGGGTGAAAGG
il17c-rat-F	GCTAACTCGAAGTGCCAGGT
il17c-rat-R	GCGGATGAACTCAGTGTGGA
fosb-rat-F	TGTGAGGACCCCTTGACTCT
fosb-rat-R	TCAGTCGGGGGTTCAATTCG
ccr9-rat-F	TGAAGCTGACTGGCGTCTGA
ccr9-rat-R	AGAGGCGGAAGGAAATGACT
nox4-rat-F	TGTTGGGCCTAGGATTGTGT
nox4-rat-R	CACTGAGAAGTTCAGGGCGT
EIF5-human-F	GCGAGAACATTCCAGAGGTC
EIF5-human-R	CATAAACCCAACGCTGCTCG
MALAT-F	AAAGTCCGCCATTTTGCCAC
MALAT-R	GCTTCATCTCAACCTCCGTCA
FOXD3-AS1-F	GGTGGAGGAGGCGAGGATG
FOXD3-AS1-R	AGCGGACAGACAGGGATTGG
Lnc-OGD1006-F	ACGTGTCTTGAGATGCCAAA
Lnc-OGD1006-R	TCCTCTCCCTCTTCCTCTCTC

### RNA-Seq

The bulk RNA-seq analysis (contract ID: 80-1220563257) was supported by AZENTA Company, Suzhou, China. The libraries for sequencing were generated from the total RNA of rat brains infected with either AAV-con or AAV-LINC00894. Approximately, 1 µg total RNA was used for library preparation.

The oligo(dT) beads were used to isolate poly(A) mRNA. First-strand cDNA and second-strand cDNA were synthesized using random primers. The purified double-stranded cDNA was then treated to repair both ends, and a dA-tail was added in one reaction, followed by T-A ligation to add adaptors at both ends. Size selection of adaptor-ligated DNA was then performed using DNA clean beads. Each sample was then amplified using PCR with P5 and P7 primers, and the PCR products were validated. Libraries with different indices were multiplexed and loaded on an Illumina HiSeq instrument (Illumina, CA, USA) and sequenced using a 2 × 150 paired-end (PE) configuration according to the manufacturer’s instructions.

### Western Blot Analysis

Cells attached in the culture wells were lysed for 30 min at 4 °C with RIPA buffer (Merck Millipore) containing 1 mM sodium orthovanadate, 100 mM NaCl, 2.5 mM Tris-HCl (pH 7.5), 10 µg/mL leupeptin, and 10 µg/mL aprotinin. The supernatant was collected from the cell lysis solution by centrifugation at 13,500 × *g* for 15 min at 4 °C and the protein concentration was measured using the Lowry protein assay. Thereafter, 15–80 µg protein was separated using PAGE and electro-transferred to a polyvinylidene fluoride membrane. The membrane was blocked by incubation with 5% nonfat milk powder in Tris-buffered saline containing tween 20 (TBST) for 2 h at 25 °C before incubation with primary antibodies at 4 °C overnight. Horseradish peroxidase-conjugated anti-mouse (1:5000; ORIGENE, China) or anti-rabbit immunoglobulin IgG (1:5000; ORIGENE) was used as the secondary antibody, and the membrane was incubated for 2 h at 25 °C. Immunoreactive bands were visualized via enhanced chemiluminescence (ECL; Bio-Rad). For quantification, the ECL signals were digitized using the Image J software (v1.50i). The antibody used were anti-EIF5 antibody (#ab170915, Abcam), anti-β-Actin antibody (#A5441, Sigma-Aldrich), anti-GAPDH antibody (#ab181602, Abcam), monoclonal antibody recognizing Ubiquitin (P4D1) (#3936, CST), Anti-ATF4 (#11,815, CST), Cleaved Caspase-3 Antibody (#9661,CST), Caspase-3 Antibody (#9662,CST), Anti-GCLC antibody (#ab207777, Abcam), Anti-IRG1 antibody (#ab222411, Abcam), Anti-FGF21 antibody (#ab171941, Abcam). All original gel/blot images are provided in Additional File 1.

### Luciferase Reporter Assay

Cells were plated in 24-well plates for 24 h and then transfected with luciferase vectors (200 ng) with or without 50 ng of ACOD1 or FGF21 expressing vector (when necessary) using Lipofectamine 3000 Reagent or jetPRIME^®^ (Polyplus Transfection, Strasbourg, France). The phRLMLP *Renilla* luciferase expression vector was co-transfected at 40 ng for each well to evaluate transfection efficiency. The cells were not lysed until 24 h post-transfection, and luciferase activity was determined using the dual luciferase reporter assay system (Promega, WI, USA). The relative promoter activity was calculated as a normalized firefly/Renilla ratio.

### ChIP Assay

BE(2)-M17 neuroblast cell line (M17) cells fixed in formaldehyde were added to the culture medium at a final concentration of 1% and maintained in 10-cm culture plates at 25 °C. The cells were shaken for 10 min before adding glycine (0.125 M). After 10 min, the cells were washed twice with cold PBS, centrifuged at 500 × *g*, and lysed in SDS lysis buffer containing 1 mM phenylmethylsulfonyl fluoride, 2 mg/mL pepstatin A, and 2 mg/mL aprotinin. Sonication was used to break the DNA into 500–1000 bp fragments. The chromatin was incubated with agarose beads containing control anti-serum or anti-ATF4 antibody (rabbit mAb, CST11815) overnight at 4 °C. The agarose beads were subsequently pelleted and washed once with a low-salt wash buffer, high-salt wash buffer, and LiCl wash buffer, and twice with TE buffer. Thereafter, the DNA bound with agarose beads and antibodies was recovered using phenol/chloroform extraction and ethanol precipitation. The eluted DNA was analyzed using PCR with the PCR products purified and sequenced.

### Statistical Analysis

Data are presented as mean ± standard error of the mean. All statistical analyses were conducted using Prism 8 (Version 8.0.1; GraphPad Software, San Diego, CA, USA). Unless otherwise mentioned, Student’s *t*-tests were used for comparisons, and results with *P* < 0.05 were considered significant.

## Results

### LINC00894 Interacted with EIF5

**Fig. 1:**
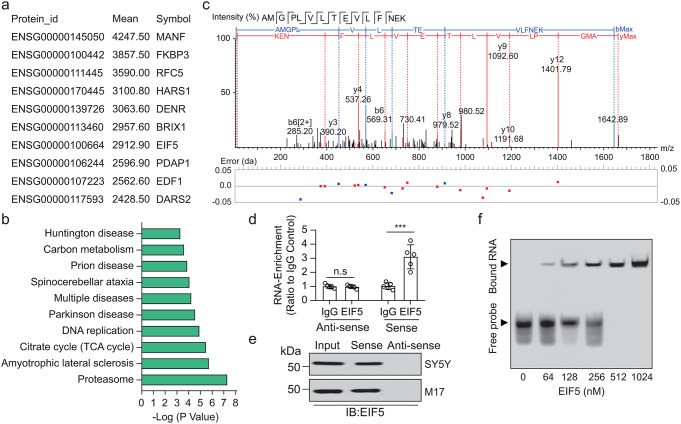
Eukaryotic translation initiation factor 5 (EIF5) was identified to interact with LINC00894. (**a**) The top 10 proteins corresponding to peptide molecular ions (> 7 amino acid) were identified from cellular components binding to biotin-labeled sense LINC00894 on mass spectrometry analysis. (**b**) GO term enrichment analysis of the top 284 identified proteins uniquely binding to LINC00894. (**c**) The cell protein lysate, upon interacting with synthetic LINC00894, exhibited molecular ion peaks characteristic of EIF5, as identified by mass spectrometry. (**d**) qPCR analysis of LINC00894 in IgG and EIF5 antibody-captured RNA–protein complex in RNA immunoprecipitation(*n* = 5); sense and anti-sense, in vitro synthesized sense and anti-sense strands of LINC00894, respectively; ***P < 0.001; n.s, no significant difference. (**e**) The representative immunoblot result showing that the in vitro translated sense LINC00894 exhibited physical interaction with EIF5. (**f**) Electrophoretic mobility shift assay was conducted using recombinant EIF5 and labeled LINC00894. The results show the interaction of EIF5 with LINC00894 in a dose-dependent manner

For discovering the proteins that interact with LINC00894, we conducted RNA-pull down experiments and obtained cellular protein components that bond to the in vitro translated sense LINC00894 and antisense LINC00894; then the sense LINC00894 and antisense LINC00894 binding proteins were identified by mass spectrometry (Fig. 1a). According to the abundance of peptide molecular ions, 284 proteins were identified to be bound to LINC00894, with the top 10 being MANF, FKBP3, RFC5, HARS1, DENR, BRIX1, EIF5, PDAP1, EDF1, and DARS2. The identified proteins were used to conduct a Gene Ontology (GO) term enrichment analysis (https://david.ncifcrf.gov/), and these proteins were mainly associated with proteasome, amyotrophic lateral sclerosis, citrate cycle, DNA replication, Parkinson’s disease, spinocerebellar ataxia, prion disease, carbon metabolism, and Huntington disease (Fig. 1b). Mass spectrometry revealed molecular ion peaks of the specific peptides of EIF5 (Fig. 1c). Therefore, we further designed experiments to verify the interaction between LINC00894 and EIF5. RNA immunoprecipitation revealed that EIF5 captured by EIF5 antibodies but not IgG control antibody, significantly enriched more sense LINC00894 than antisense LINC00894 (Fig. 1d). The immunoblot assay confirmed that the in vitro translated sense LINC00894 physically interacted with EIF5 (Fig. 1e). In addition, EIF5 also interacted with LINC00894 in a dose-dependent manner, as determined using EMSA (Fig. 1f). These results demonstrated that LINC00894 could be a participant in cellular metabolism and neuron dysfunction.

### LINC00894 and EIF5 Interaction Promoted EIF5 Stabilization

**Fig. 2:**
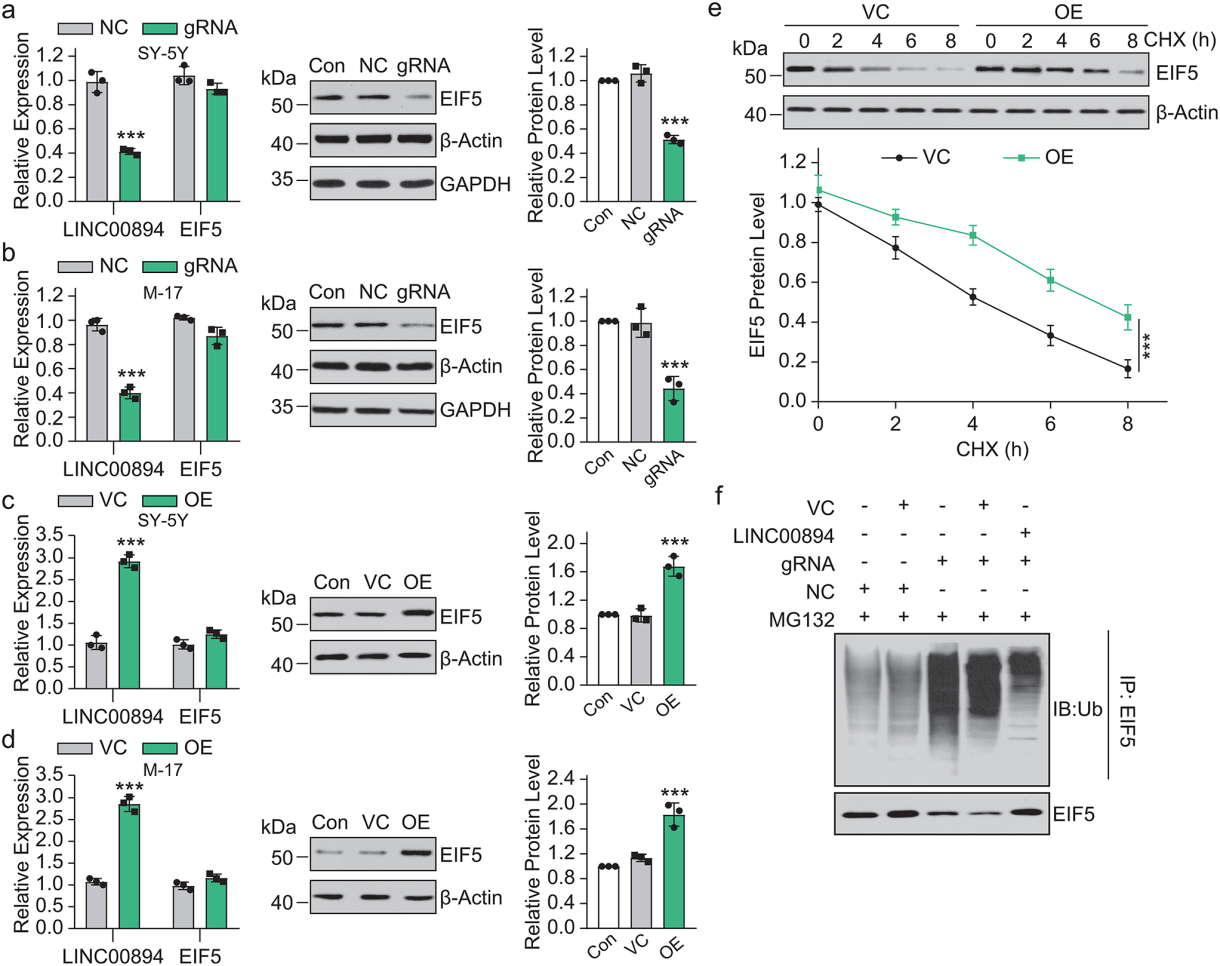
LINC00894 and EIF5 interaction promoted EIF5 stabilization. (**a–b**) The effect of LINC00894 knockdown using CRISPR/Cas9-mediated gene editing on EIF5 mRNA and protein expression in (**a**) SH-SY5Y and (**b**) M-17 cells(*n* = 3). ***, NC vs. gRNA, *P* < 0.001. ANOVA test was used to compare the difference between indicated groups. (**c-d**) The effect of LINC00894 overexpression on EIF5 mRNA and protein expression in (**c**) SH-SY5Y and (**d**) M-17 cells(*n* = 3). ***, VC vs. OE, *P* < 0.001, ANOVA test was used to compare the difference between indicated groups. (**e**) Immunoblot showing the effect of LINC00894 overexpression on the EIF5 degradation rate in cells with cycloheximide (10 µM)-induced inhibition of de novo protein synthesis(*n* = 3). ***, OE (green) vs. VC (black) at 8 h, *P* < 0.001. (**f**) Immunoprecipitation using EIF5 antibody, followed by immunoblotting using ubiquitin antibody, showed that LINC00894 knockdown increased ubiquitination modification of EIF5, whereas restoring the expression of LINC00894 decreased ubiquitination modification of EIF5. NC, control vector of gene edit; gRNA, gene edit vector targeting LINC00894; VC, empty vector control; OE, LINC00894 overexpression vector; MG132, the peptide-aldehyde proteasome inhibitor carbobenzoxyl-L-leucyl-L-leucyl-L-leucine

Because in an RNA expression correlation analysis of human samples suggested that LINC00894 and EIF5 are co-expressed and may together regulate long-term potentiation (LTP) dysfunction [34], we decided to investigate the impact of LINC00894 expression levels on EIF5 protein expression. LINC00894 knockdown mediated by CRISPR/Cas9 system [38] did not change the *EIF5* mRNA expression but decreased the EIF5 protein level in M17 or SH-SY5Y cells (Fig. 2a, b). In contrast, the overexpression of LINC00894 increased the EIF5 protein level without changing the *EIF5* mRNA expression in the two cell lines (Fig. 2c, d).

The overexpression of LINC00894 substantially decreased the degradation rate and increased the half-life of EIF5 (Fig. 2e). Meanwhile, LINC00894 knockdown increased the ubiquitination modification of EIF5; restoring the expression of LINC00894 decreased the ubiquitination modification of EIF5 (Fig. 2f). Thus, we believed that the binding of LINC00894 to EIF5 promoted EIF5 stabilization by inhibiting the ubiquitination of EIF5.

### Ectopic Expression of LINC00894 Protected the Brain Against Injury in MCAO Animals Model

For cells lacking EIF5 display an imbalance in cell proliferation and apoptosis in *Drosophila* [26], and LINC00894 regulated cellular EIF5 level (Fig. 2) and could participant in neuron dysfunction (Fig. 1), we hypnotized that LINC00894 could regulate cell proliferation and apoptosis in brain. We utilized the middle cerebral artery occlusion reperfusion (MCAO/R) model to investigate whether LINC00894 affects brain tissue damage in a brain ischemia model.

**Fig. 3:**
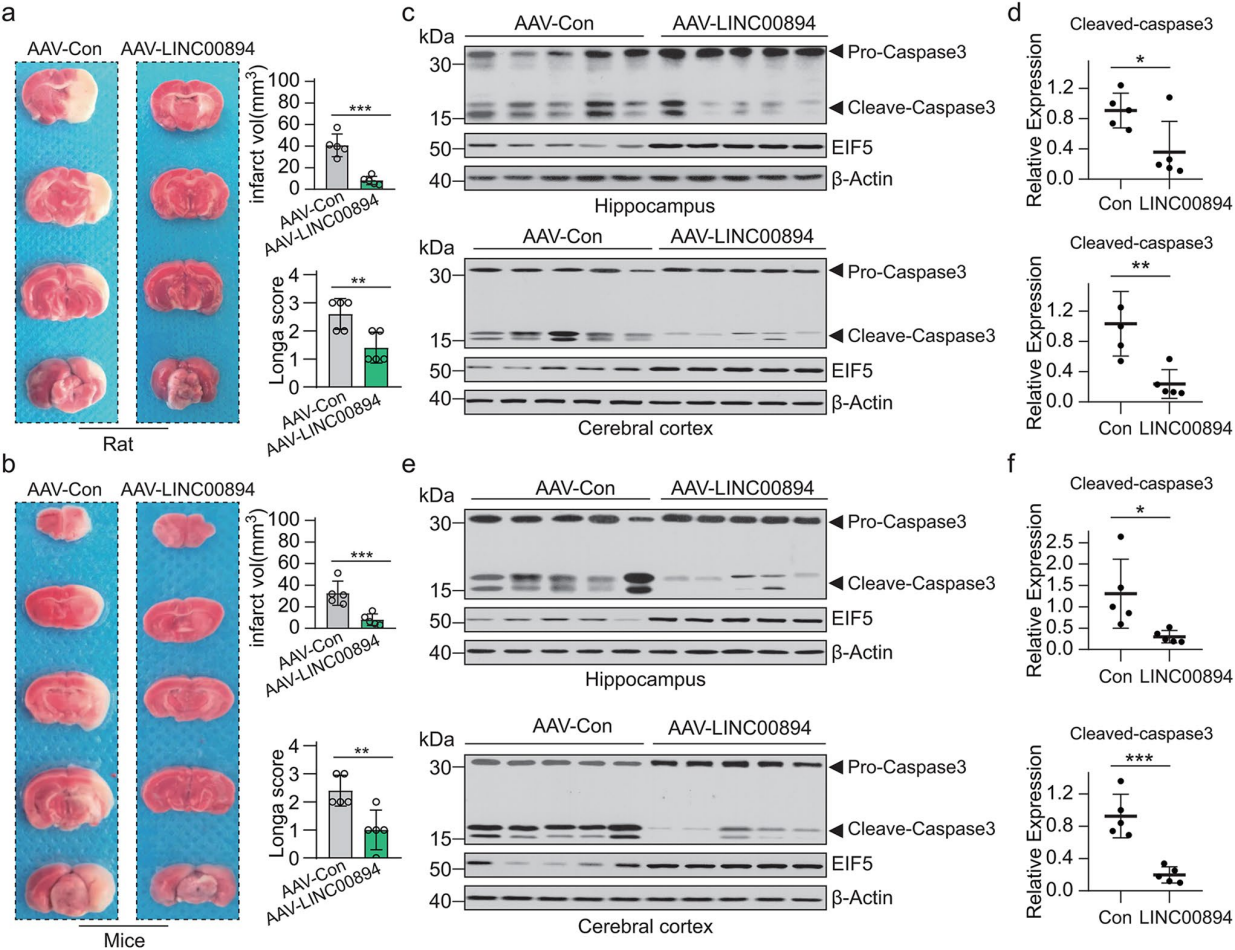
Ectopic expression of LINC00894 protects against brain injury in MCAO mice. (**a–b**) The representative cerebral infarct volume of rats (**a**) and mice (**b**) after cerebral ischemia/reperfusion injury *via* MCAO with TTC staining in control virus (AAV-con) and virus carrying LINC00894 gene (AAV-LINC00894) groups, with statistical results of cerebral infarct volume and neurological behavioral test by longa neurological examination score in each model (*n* = 5). Infarct volumes were quantified using the Image-Pro-Plus v.6.0 software (right panel). ***, AAV-con vs. AAV-LINC00894, *P* < 0.001. (**c–f**) Immunoblot showing the expression of activated caspase-3 and EIF5 in hippocampus and cerebral cortex from rat (**c**) and mice (**e**) in the indicated groups (*n* = 5), respectively, with quantification of relative expression of activated caspase-3 in rat (**d**) and mice (**f**). AAV-con, control virus infected tissues; AAV-LINC00894, virus carrying LINC00894 gene infected tissues. AAV-con vs. AAV-LINC00894, *P < 0.05; **P < 0.01; *** *P* < 0.001

Compared to AAV-con, AAV-LINC00894 significantly reduced the infarct volume at 24 h after MCAO based on 2,3,5-triphenyltetrazolium chloride (TTC) staining (Fig. 3a, b). The longa neurological examination score was used to evaluated the effect LINC00894 overexpression on neurological deficits in the MCAO models (Fig. 3a, b). The Results showed that AAV-LINC00894 significantly reduced the longa scores, indicating the LINC00894 overexpression reduced neurological damage of the rats and mice in the MCAO model.

Furthermore, the western blot analysis demonstrated that the ectopic expression of LINC00894 significantly inhibited the expression of activated caspase-3 in the hippocampus and cerebral cortex of rats (Fig. 3c, d) and mice (Fig. 3e, f). These results indicated that LICN00894 inhibited brain cell apoptosis and attenuated brain tissue damage in the MCAO model.

### LINC00894 Protected Against OGD-Induced Cell Apoptosis

**Fig. 4:**
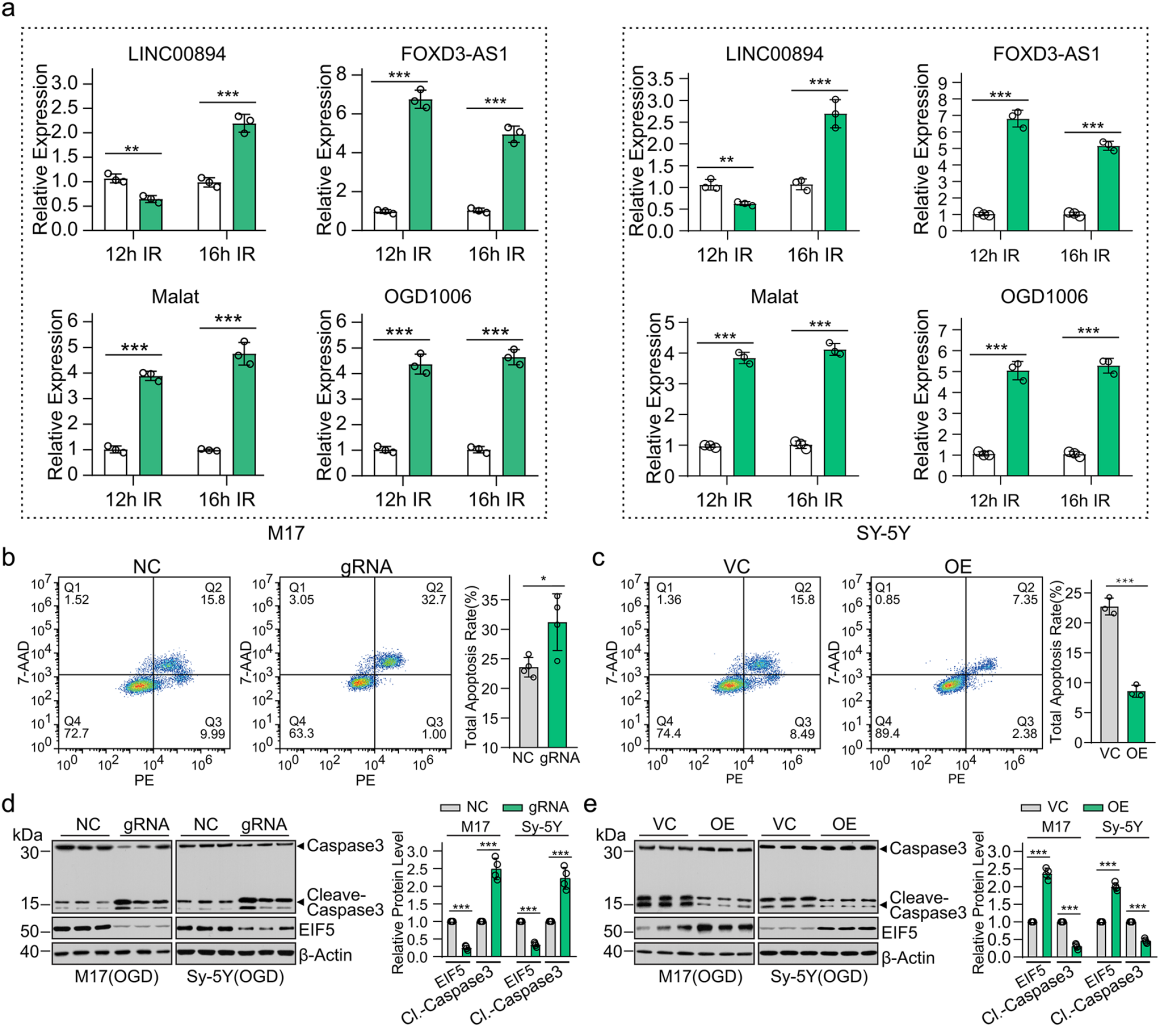
LINC00894 protects against oxygen–glucose deprivation-induced cell apoptosis. (**a**) qRT-PCR was used to determine the effect of OGD challenge for the indicated time on the expression of Malat, FOXD3-AS1, and OGD1006 in M-17 and SH-SY5Y cells (*n* = 3). *** *P* < 0.001; n.s., no significant difference. (**b-e**) Representative results of apoptosis analysis in indicated treatment with the gates and regions placed around populations of cells with PE and 7-AAD staining, with the ratio of apoptotic cells quantified in column in M17 cells challenged with 16 h of OGD (**b**, *n* = 4; **c**, *n* = 3); immunoblots showing the effects of LINC00894 knockdown (**d**) (*n* = 4) or overexpression (**e**) (*n* = 4) on the expression of activated Caspase-3, with the quantification in column, in M-17 and SH-SY5Y cells; β-actin was used as the loading control; NC, empty gene edit vector lentiCRISPR v2 (Addgene:52,961); gRNA, lentiCRISPR v2 vector carrying U6-gRNA targeting LINC00894; VC, pcDNA3.1 vector; OE, pcDNA3.1 carrying LINC00894 clone. Cl.-Caspase3, Cleaved-Caspase3;

We further evaluated the effects of OGD on the expression of LINC00894, Malat, FOXD3-AS1, and OGD1006 by qRT-PCR in M-17 and SH-SY5Y cells. The results showed that in both M17 and SH-SY5Y cells, the expression of Malat, FOXD3-AS1, and OGD1006 was significantly increased after 12–16 h of OGD exposure, whereas the expression of LINC00894 was first numerically decreased after 12 h of OGD exposure, but later after 16 h of OGD exposure it was significantly increased, indicating that LINC00894 may be a potential stress response gene (Fig. 4a).

The subsequent experiment conducted using the flow cytometry-based method demonstrated that LINC00894 overexpression significantly inhibited, whereas LINC00894 knockdown significantly promoted, cell apoptosis induced by 16 h of OGD challenge in M17 cells (Fig. 4b, c). The immunoblot analysis further demonstrated that LINC00894 knockdown significantly enhanced, whereas LINC00894 overexpression significantly suppressed, the expression of activated caspase-3 (Fig. 4d, e). The results obtained in these in vitro models indicated that LINC00894 protected against OGD-induced cell apoptosis.

### **LINC00894-Stabilized EIF5 Increased ATF4 Expression**, **Affecting Cellular GSH Level**

**Fig. 5:**
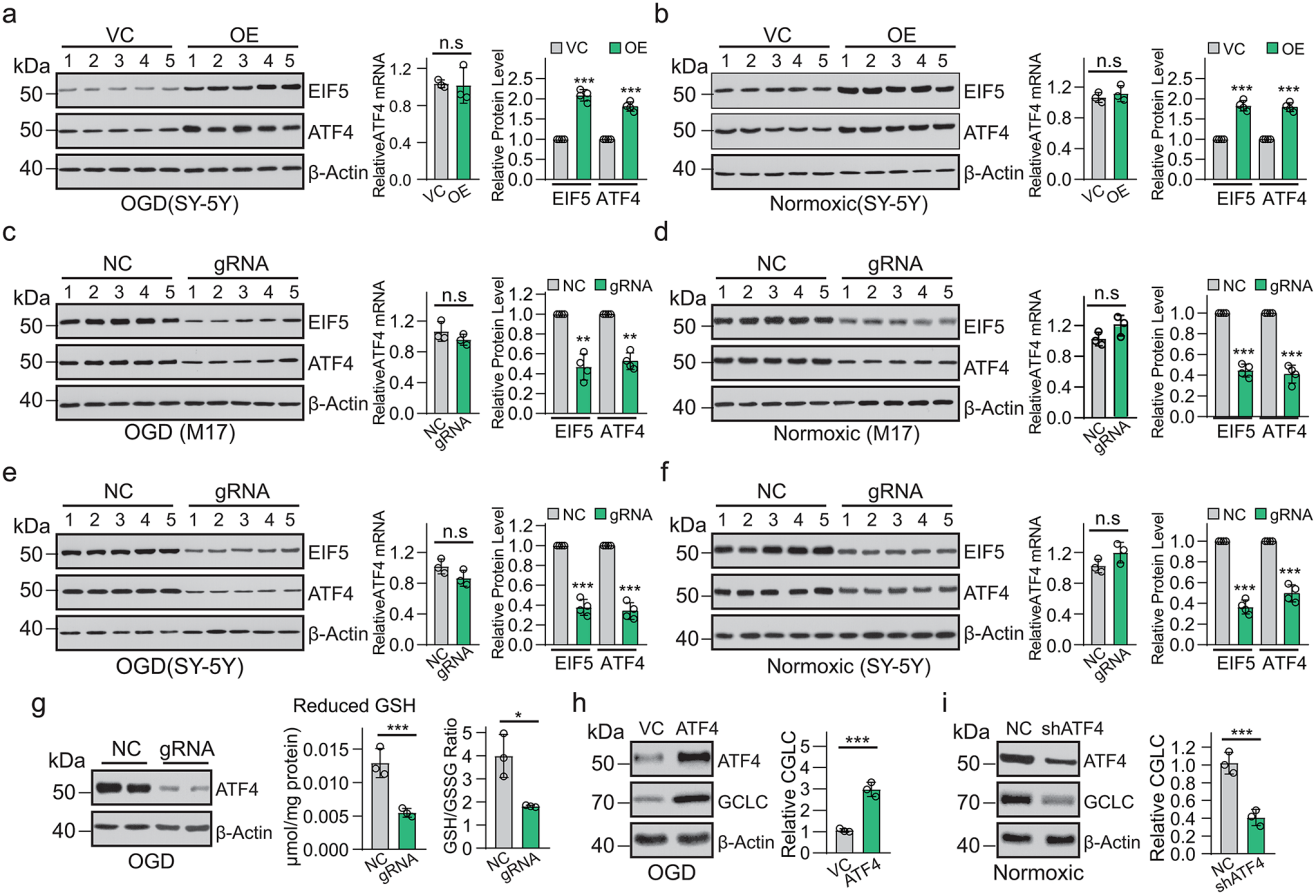
LINC00894 stabilizes ATF4 expression, affecting the cellular glutathione level. (**a**, **b**) Immunoblot showed the effect of ectopic expression of LINC00894 on indicated protein expression (left), with representative ATF4 mRNA expression analysis (middle column, *n* = 3) and quantification of the immunoblots (right column, *n* = 4) in SH-SY5Y cells under 16 h of OGD challenge (**a**) or normoxic (**b**); each treatment in quintuplicate were repeated 4 times with cells collected for analysis (immunoblot) at 24 h of treatment (OGD or normoxic); (**c–f**) Immunoblot showed the effect of LINC00894 knockdown on indicated protein expression (left), with representative ATF4 mRNA expression analysis (middle column, *n* = 3) and quantification of the immunoblots (right column, *n* = 4) in M17 (**c**, **d**) and SH-SY5Y (**e**, **f**) cells under the condition of OGD challenge (**c**, **e**) or normoxic (**d**, **f**); each treatment in quintuplicate were repeated 4 times with cells collected for immunoblot at 24 h of treatment (OGD or normoxic); (**g**) the effect of LINC00894 knockdown on cellular ATF4 protein expression, GSH level, and GSH: GSSG ratio in SH-SY5Y under the condition of OGD challenge for 16 h (*n* = 3). (**h**) Overexpression of ATF4 increases CGLC expression at 16 h in OGD-induced SH-SY5Y cells (*n* = 3). (**i**) ATF4 knockdown increases CGLC expression in SH-SY5Y cells under normoxia (*n* = 3). n.s, no significant difference; *P < 0.05; *** *P* < 0.001

For eukaryotic translation initiation factor 5 is critical for accurate control of translation of GCN4 [23], the yeast ATF4 equivalent [24], we further investigate whether LINC00894 promoting EIF5 stability would affect the ATF4 expression. In the immunoblot assay, the ectopic expression of EIF5 increased the ATF4 expression but not *ATF4* mRNA in SH-SY5Y cells under both OGD condition and normal condition (normoxic) (Fig. 5a, b). *EIF5* knockdown decreased ATF4 protein expression but not *ATF4* mRNA expression in SH-SY5Y and M17 cells under both OGD condition and normal condition (normoxic) (Fig. 5c-f).

Because ATF4 is involved in promoting protein synthesis and stimulating cellular cystine uptake and glutathione (GSH) synthesis [13, 14]. We further investigated whether LINC00894 knockdown would affect the cellular GSH level. LINC00894 knockdown led to a decrease in cellular ATF4 protein and GSH levels and the GSH: GSSG ratio (Fig. 5g) in SH-SY5Y under OGD conditions. The overexpression of LINC00894 also demonstrated an increase in glutamate-cysteine ligase catalytic subunit (CGLC) expression in OGD-induced SH-SY5Y cells (Fig. 5h), whereas LINC00894 knockdown decreased cellular ATF4 and CGLC protein expression (Fig. 5f). These results demonstrated that LINC00894 stabilized EIF5 and could promote ATF4 translation, resulting in the regulation of CGLC expression and GSH synthesis.

### LINC00894 Overexpression Increased ATF4 Target FGF21 and ACOD1 Expression

To investigate the mechanism of LINC00894 protecting against stress induced cells apoptosis in OGD and MCAO model, we started to investigate the LINC00894 potentially regulated ATF4 target genes in brain from MCAO model.

**Fig. 6:**
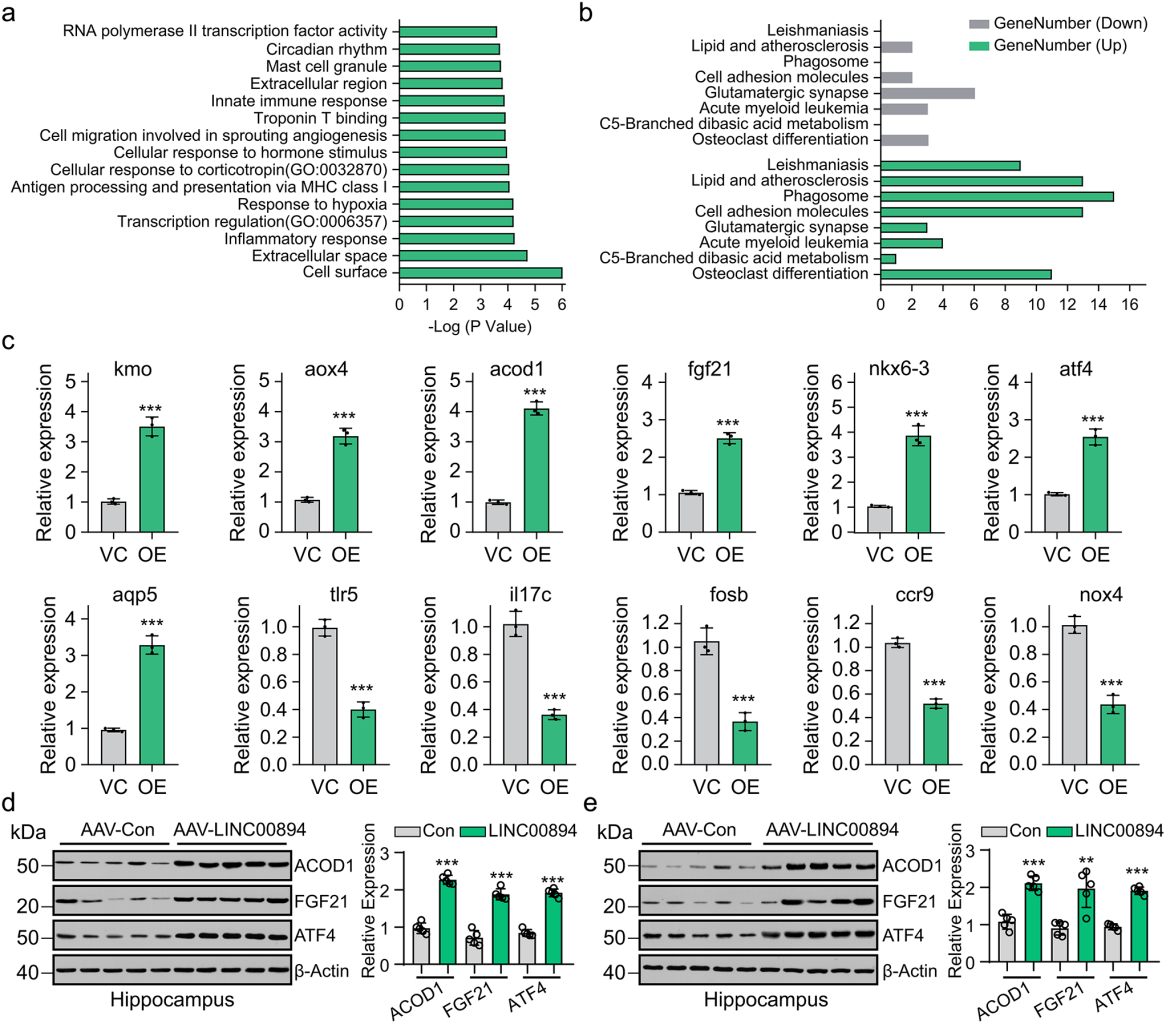
AAV virus-mediated LINC00894 overexpression regulates FGF21 and ACOD1 expression in rat and mouse hippocampus. (**a**) GO and (**b**) KEGG analysis of the differentially expressed genes from RNA-seq conducted using AAV-con- (*n* = 3) and AAV-LINC00894-infected whole brains (*n* = 3). (**c**) qPCR was used to detect the expression of ATF4 target genes that differ from those in RNA-seq in PC12 cells subjected to oxygen–glucose deprivation challenge for 12 h (*n* = 3). The representative result showed that overexpression of LINC00894 increased the protein expression of ATF4, FGF21, and ACOD1 in the hippocampus of the (**d**) rats(*n* = 5) and (**e**) mice (*n* = 5) in the MCAO model, with quantification of indicated bands in each group from 3 immune blot assay. *** *P* < 0.001

RNA-Seq (GSE268399) was used to compare gene expression differences between AAV-con- and AAV-LINC00894-infected brains. TOP120 genes mostly regulated by LINC00894 in rats brain from MCAO model in RNA-seq assay was shown in ‘Additional File 2’. The GO analysis revealed that LINC00894 mainly regulated genes related to the inflammatory response, transcription regulation (GO:0006357), response to hypoxia (GO:0001666), antigen processing and presentation via MHC class I (GO:0042590), cellular response to corticotropin (GO:0032870), cellular response to hormone stimulus (GO:0032870), cell migration involved in sprouting angiogenesis (GO:0002042), troponin T binding (GO:0031014), and innate immune response (GO:0045087) and circadian rhythm (GO:0007623) (Fig. 6a). The Kyoto Encyclopedia of Genes and Genomes (KEGG) pathway analysis (https://david.ncifcrf.gov/) revealed that the LINC00894-regulated genes mainly affected osteoclast differentiation, C5-branched dibasic acid metabolism, acute myeloid leukemia, glutamatergic synapse, phagosome, lipid, and atherosclerosis (Fig. 6b).

From the top 120 affected genes regulated by LINC00894 (Additional File 1), we selected *kmo*, *aox4*, *acod1*, *fgf21*, *nkx6-3*, *atf4*, *aqp5*, *tlr5*, *il17c*, *fosb*, *ccr9*, and *nox4* as the potential ATF4 target genes or those participating in LINC00894-mediated protection against MCAO. Using qRT-PCR, it was determined that the expression of these genes was also regulated by overexpressing LINC00894 in PC12 cells subjected to ODG (Fig. 6c). Furthermore, LINC00894 overexpression increased the protein expression of ATF4, FGF21, and ACOD1 in the hippocampus of MCAO rats and mice (Fig. 6d, e). Based on these findings, we believed that AAV-mediated ectopic expression of LICN00894 could affect FGF21 and ACOD1 expression, through which the protective effect against ischemia-induced brain injury is exercised.

### LINC00894-Mediated Protection Against Neuronal OGD Injury Depends on ATF4 Transcriptionally Regulating FGF21 and ACOD1 Expression

**Fig. 7:**
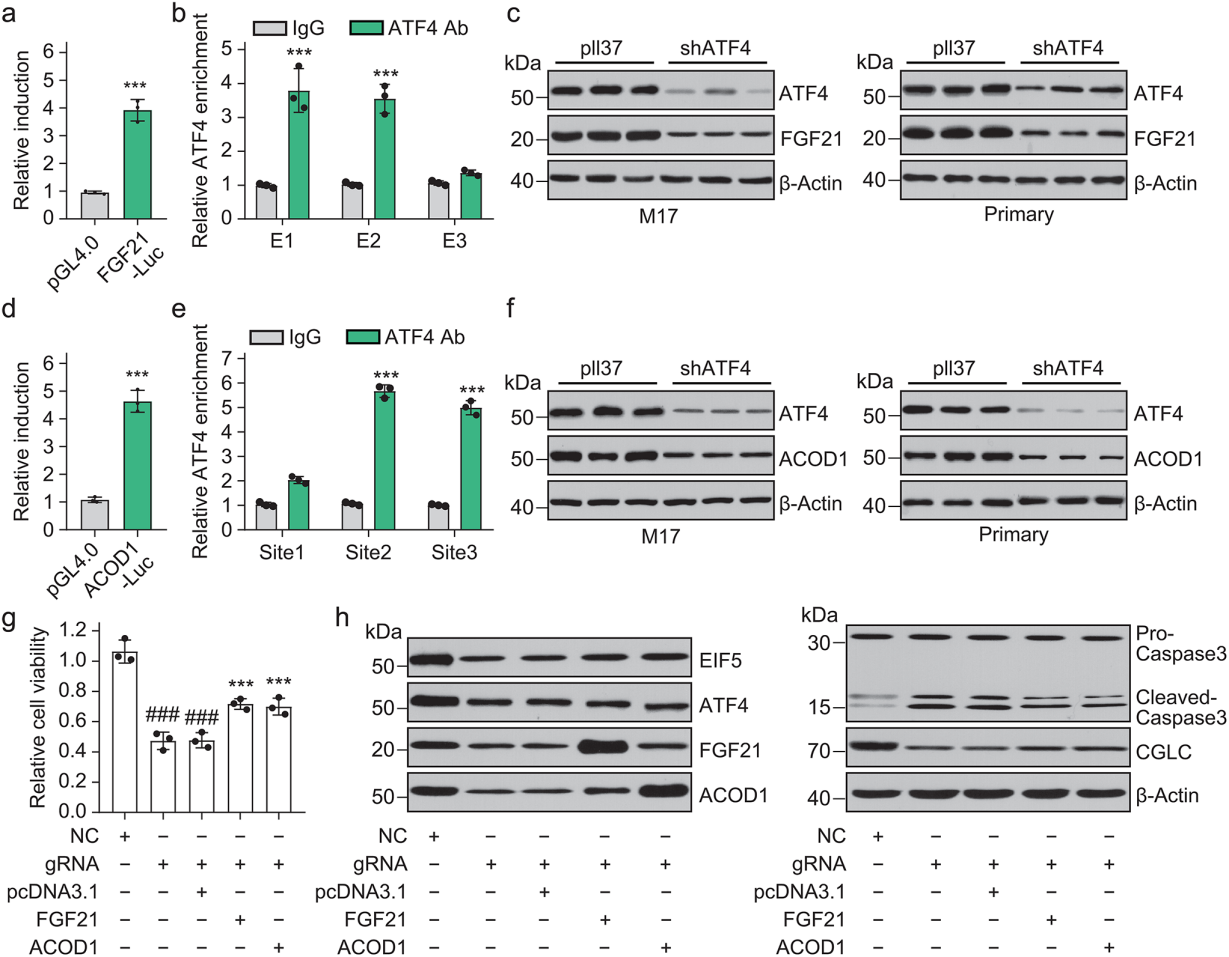
LINC00894-mediated protection of neuron from OGD depends on ATF4 transcriptionally regulating FGF21 and ACOD1 expression. (**a**) ATF4 increases the luciferase activity of the FGF21 reporter gene; pGL4.0, empty control vector for luciferase assay; FGF21-Luc, FGF21 luciferase vector (*n* = 3). (**b**) Chromatin immunoprecipitation (ChIP) to evaluate the binding of ATF4 to its consensuses (E1, E2, and E3) in the FGF21 promoter (*n* = 3). (**c**) The representative result showed the effect of *ATF4* knockdown mediated by shRNA on FGF21 expression in M17 (left) and primary fibroblast cells (right). (**d**) ATF4 increases the luciferase activity of the ACOD1 reporter gene; pGL4.0, empty control vector for luciferase assay(*n* = 3); ACOD1-Luc, ACOD1 luciferase vector. (**e**) ChIP was performed to evaluate the binding of ATF4 to its consensuses (site1, site2, and site3) in the ACOD1 promoter(*n* = 3). (**f**) The representative result showed the effect of *ATF4* knockdown mediated by shRNA on ACOD1 expression in M17 (left) and primary fibroblast cells (right). (**g**, **h**) Forced expression of FGF21 or ACOD1 in LINC00894-knockdown M17 cells restored cell viability (**g**) as assessed using CCK-8(*n* = 3) and inhibited activated caspase-3 expression (**h**) as determined in representative western blot assay

Existing findings have shown that ATF4 directly regulates the transcription of FGF21 [16]. We also found that ectopic ATF4 can increase the activity of the FGF21 reporter gene (Fig. 7a), and the ChIP analysis results also indicated that ATF4 directly binds to the transcriptional regulatory region of FGF21 (Fig. 7b). Immunoblotting confirmed that *ATF4* knockdown leads to a decrease in FGF21 protein expression in M17 cells and primary hippocampal neurons (Fig. 7c). These results demonstrated the regulation of FGF21 transcription by ATF4. We also investigated whether ATF4 transcriptionally regulated the expression of ACOD1. The results of the luciferase assay showed that ATF4 increased the activity of the ACOD1 luciferase reporter (Fig. 7d). In the ChIP assay, ATF4 was enriched in the ACOD1 promoter (Fig. 7e). The immunoblot assay showed that *ATF4* knockdown decreased ACOD1 expression in both M17 and primary hippocampal neurons (Fig. 7f). Thus, ATF4 could transcriptionally regulate ACOD1 expression.

We further investigated whether LINC00894-mediated protection of neurons from OGD depended on ATF4, which transcriptionally regulates FGF21 and ACOD1 expression. In the restoration experiment, we found that restoring the expression of FGF21 or ACOD1 in LINC00894-knockdown cells increased cell viability (Fig. 7g) and decreased activated caspase-3 expression in the OGD/R model (Fig. 7h). Thus, we confirmed that LINC00894 is a stress response gene that protects against cerebral ischemic injury by stabilizing EIF5 and facilitating EIF5-ATF4-dependent induction of FGF21 and ACOD1 expression.

## Discussion

Approximately 40 million disabilities worldwide are caused by cerebral ischemia annually [39]. Thus, studying the causes and mechanisms of cerebral ischemic stroke is of importance. Although some studies have indicated that LINC00894 may affect LTP function and GPRIN1 expression, shedding light on its potential function in the brain [34, 35], in this study, we demonstrated that this RNA protected the brain from ischemic injury in animal and cell culture models and partially elucidated the molecular mechanisms underlying the role of LINC00894 in this process.

The results of the interactome analysis revealed that LINC00894 may interact with FKBP3, DENR, and DARS2 (Fig. 1a). FKBP3, encoding FKBP25, likely regulates ribosome biogenesis and interacts with the 60 S ribosomal protein L7a; FKBP25 protects endothelial cells against OGD injury [40]. De novo mutations in DENR detected in humans impair its function in mRNA translation and disrupt the migration and terminal branching of cortical neurons [41], DARS2, encoding aspartyl-tRNA synthetase 2, protects against neuroinflammation [42] and regulates the initial stages of mitochondrial protein production [43]. As FKBP3, DENR, DARS2, and EIF5 can regulate protein biosynthesis and bind to LINC00894 in the interactome, we deduced that LINC00894 might regulate protein biosynthesis via interaction with multiple functional molecules; thus, it can exercise neuron protection via other signaling mechanisms.

Animals carrying EIF5G31R-mutant cells reportedly showed low GSH levels, high ROS activity, and H_2_O_2_ sensitivity [44]. Cyst stem cells lacking EIF5 display an imbalance in cell proliferation and apoptosis in *Drosophila* [26]. Consistent with these findings, the cellular EIF5, which was stabilized by LINC00894 in immortal cells (Figs. 4 and 5) and brain tissues (Fig. 3), exhibited protective effects against ischemia (Fig. 3), further demonstrating that EIF5-controlled translation would benefit cellular survival (Figs. 3 and 4). EIF5 stabilized by LINC00894 facilitated GSH synthesis (Fig. 5g) [44], protecting brain cells from oxidative stress, and induced apoptosis during ischemic injury [45]. In addition, the findings of this study are consistent with those of previous studies; furthermore, our findings demonstrated that EIF5 controls the translation of *ATF4* (Fig. 5) [23–25]. Combing all these findings we would propose a hypothesis that LINC00894 could regulate ATF4 translation (Fig. 5d) via affecting ubiquitination mediated degradation of EIF5 (Fig. 2e, f).

In this study, we demonstrated that LINC00894 regulates the expression of *ATF4* (Figs. 5 and 6) and *GCLC* and affects the cellular levels of GSH (Fig. 5g–i). Therefore, it is believed that LINC00894 can regulate cellular oxidative stress, thus strengthening our understanding of *ATF4* as a stress-responsive gene regulating oxidative stress and GSH synthesis [13, 14]. Furthermore, the expression of FGF21 and ACOD1 via ATF4-mediated transcriptional regulation (Fig. 7a–f) could be modulated by LINC00894, and the protective role of LINC00894 in ischemic injury depends on the levels of intracellular FGF21 and ACOD1 (Fig. 7g, h). These findings indicate that ATF4 directly regulates FGF21 expression in multiple biological contexts and show that ATF4 directly regulates ACOD1 signaling.

By catalyzing itaconate production, ACOD1 regulates oxidative stress and antigen processing and plays dual roles in inflammation [27, 46]. ACOD1-catalyzed itaconate inhibits succinate dehydrogenase activity, resulting in succinate accumulation. Excess succinate inhibits the expression of proinflammatory genes by impairing mitochondrial ROS production; In contrast, ACOD1-mediated ROS production leads to the induction of proinflammatory cytokines [46, 47]. In this study, LINC00894 regulated ACOD1 expression in an *ATF4-*dependent manner to inhibit ischemia injury-induced cellular apoptosis, indicating that LINC00894-regulated ACOD1 expression could have an anti-inflammatory effect as reported previously [28].

In conclusion, LINC00894 could exert biological effects by interacting with EIF5. Using MCAO and in vitro ischemia models, we showed that LINC00894 stabilized EIF5 to enhance the expression of ATF4, which transcriptionally regulated the expression of FGF21 and ACOD1, thus protecting cells from brain ischemia-induced damage. This study highlights the potential importance of LINC00894 in translation regulation, oxidative stress, and inflammatory responses. The limitation of this study is that we did not fully elucidate the mechanism by which LINC00894 regulates EIF5 ubiquitin degradation, as well as the precise role of ubiquitination of EIF5 in inflammatory responses. Further research is needed to determine which specific cell types in the animal brain are primarily affected by LINC00894 and how it exerts a protective effect on their functionality.

### Electronic Supplementary Material

Below is the link to the electronic supplementary material.

Supplementary Material 1: Original gel/blot images. All original gel/blot images corresponding to the manuscript figures.

Supplementary Material 2: Supplementary Table 1. TOP120 genes mostly regulated by LINC00894 in mice brain from MCAO model in RNA-seq assay.

## Data Availability

No datasets were generated or analysed during the current study.

## Abbreviations

AAV: Adeno-associated virus
ACOD1: Aconitate decarboxylase 1
ATCC: American Type Cell Culture Collection
ATF4: Activating transcription factor 4
ChIP: Chromatin immunoprecipitation
CI/R: Cerebral ischemia–reperfusion
EIF5: Eukaryotic translation initiation factor 5
EMSA: Electrophoretic mobility shift assay
FGF21: Fibroblast growth factor 21
GCLC: Glutamate-cysteine ligase catalytic subunit
GCLM: γ-glutamyl-cysteine ligase synthetase including γ-GC modifier subunit
GO: Gene ontology
GSH: Glutathione
GSSG: Glutathione disulfide
lncRNA: Long non-coding RNA
LTP: Long-term potentiation
MBF: Mouse brain fibroblasts
MCAO/R: Middle cerebral artery occlusion reperfusion
OGD/R: Oxygen–glucose deprivation and reoxygenation
PBS: Phosphate-buffered saline
qRT-PCR: Quantitative reverse transcription polymerase chain reaction
ROS: Reactive oxygen species
TGF-β2: Transforming growth factor-beta 2
TTC: 2,3,5-triphenyltetrazolium chloride
ZEB1: Zinc finger E-box-binding homeobox 1
