# Supplementary material for: LINC00894 Regulates Cerebral Ischemia/Reperfusion Injury by Stabilizing EIF5 and Facilitating ATF4-Mediated Induction of FGF21 and ACOD1 Expression
Source: Neurochem Res. 2024 Jul 26;49(10):2910–25. doi: 10.1007/s11064-024-04213-w (PMC11365926; doi:10.1007/s11064-024-04213-w)
Supplement: Supplementary file 2 — Supplementary Material 2: Supplementary Table 1. TOP120 genes mostly regulated by LINC00894 in mice brain from MCAO model in RNA-seq assay. [file 11064_2024_4213_MOESM2_ESM.docx]

| Fig.1d, SY5Y，EIF5 |  |
| --- | --- |
| Fig.1d, M17，EIF5 |  |
| Fig.1e，EMSA |  |
| Fig.2a, EIF5 |  |
| Fig.2a, ꞵ-Actin |  |
| Fig.2a, GAPDH |  |
| Fig.2b, EIF5 |  |
| Fig.2b, ꞵ-Actin |  |
| Fig.2b, GAPDH |  |
| Fig.2c, EIF5 |  |
| Fig.2c, ꞵ-Actin |  |
| Fig.2d, EIF5 |  |
| Fig.2d，ꞵ-Actin |  |
| Fig.2e，EIF5 |  |
| Fig.2e，ꞵ-Actin |  |
| Fig.2f, Ub |  |
| Fig.2f, EIF5 |  |
| Fig.3c, Caspase-3(total and activated), Hippocampus |  |
| Fig.3c, EIF5, Hippocampus |  |
| Fig.3c, ꞵ-Actin, Hippocampus |  |
| Fig.3c, Caspase-3(total and activated), Cerebral cortex |  |
| Fig.3c, EIF5, Cerebral cortex |  |
| Fig.3c, ꞵ-Actin, Cerebral cortex |  |
| Fig.3e, Caspase-3(total and activated), Hippocampus |  |
| Fig.3e, EIF5, Hippocampus |  |
| Fig.3e, ꞵ-Actin, Hippocampus |  |
| Fig.3e, Caspase-3(total and activated), Cerebral cortex |  |
| Fig.3e, EIF5, Cerebral cortex |  |
| Fig.3e, ꞵ-Actin, Cerebral cortex |  |
| Fig 4d，Caspase3(total and activated)，M17 |  |
| Fig 4d，EIF5，M17 |  |
| Fig 4d，ꞵ-Actin，M17 |  |
| Fig 4d，Caspase3(total and activated)，SY-5Y |  |
| Fig 4d, EIF5，SY-5Y |  |
| Fig 4d, EIF5，SY-5Y |  |
| Fig 4e, Caspase3(total and activated)，M17 |  |
| Fig 4e, M17, EIF5 |  |
| Fig 4e, M17, ꞵ-Actin |  |
| Fig 4e，caspase3(total and activated)，SY-5Y |  |
| Fig 4e，EIF5，SY-5Y |  |
| Fig 4e， ꞵ-Actin，SY-5Y |  |
| Fig.5a, EIF5, SY-5Y,OGD |  |
| Fig.5a, ATF4, SY-5Y,OGD |  |
| Fig.5a, ꞵ-Actin, SY-5Y, OGD |  |
| Fig.5b, EIF5, SY-5Y, Normoxic |  |
| Fig.5b, ATF4, SY-5Y, Normoxic |  |
| Fig.5b, ꞵ-Actin, SY-5Y, Normoxic |  |
| Fig.5c, EIF5, M17, OGD |  |
| Fig.5c, ATF4, M17, OGD |  |
| Fig.5c, ꞵ-Actin, M17, OGD |  |
| Fig.5d, EIF5, M17, Normoxic |  |
| Fig.5d, ATF4, M17, Normoxic |  |
| Fig.5d, ꞵ-Actin, M17, Normoxic |  |
| Fig.5e, EIF5, SY-5Y, OGD |  |
| Fig.5e, ATF4, SY-5Y, OGD |  |
| Fig.5e, ꞵ-Actin, SY-5Y, OGD |  |
| Fig.5f, EIF5, SY-5Y, Normoxic |  |
| Fig.5f, ATF4, SY-5Y, Normoxic |  |
| Fig.5f, ꞵ-Actin, SY-5Y, Normoxic |  |
| Fig.5g, ATF4 |  |
| Fig.5g, ꞵ-Actin |  |
| Fig.5h, ATF4 |  |
| Fig.5h, GCLC |  |
| Fig.5h, ꞵ-Actin |  |
| Fig.5i, ATF4 |  |
| Fig.5i, GCLC |  |
| Fig.5i, ꞵ-Actin |  |
| Fig.6d, ACOD1 |  |
| Fig.6d, FGF21 |  |
| Fig.6d, ATF4 |  |
| Fig.6d, ꞵ-Actin |  |
| Fig.6e, ACOD1 |  |
| Fig.6e, FGF21 |  |
| Fig.6e, ATF4 |  |
| Fig.6e, ꞵ-Actin |  |
| Fig.7c, ATF4, M17 |  |
| Fig.7c, FGF21, M17 |  |
| Fig.7c, ꞵ-Actin, M17 |  |
| Fig.7c, ATF4, Primary |  |
| Fig.7c, FGF21, Primary |  |
| Fig.7c, ꞵ-Actin, Primary |  |
| Fig.7f, ATF4，M17 |  |
| Fig.7f, ACOD1，M17 |  |
| Fig.7f, ꞵ-Actin，M17 |  |
| Fig.7f, ATF4, Primary |  |
| Fig.7f, ACOD1, Primary |  |
| Fig.7f, ꞵ-Actin, Primary |  |
| Fig.7h, EIF5 |  |
| Fig.7h, ATF4 |  |
| Fig.7h, FGF21 |  |
| Fig.7h, ACOD1 |  |
| Fig.7h, Caspase3 |  |
| Fig.7h, CGLC |  |
| Fig.7h, ꞵ-Actin |  |
